# Blood–Brain Barrier Dysfunction Predicts Microglial Activation After Traumatic Brain Injury in Juvenile Rats

**DOI:** 10.1089/neur.2023.0057

**Published:** 2024-02-08

**Authors:** Tabitha R.F. Green, Tina Nguyen, Veronika Dunker, Danielle Ashton, J. Bryce Ortiz, Sean M. Murphy, Rachel K. Rowe

**Affiliations:** ^1^ Department of Integrative Physiology, University of Colorado Boulder, Colorado, USA.; ^2^ Department of Child Health, University of Arizona College of Medicine–Phoenix, Arizona, USA.; ^3^ Cumberland Biological and Ecological Researchers, Longmont, Colorado, USA.

**Keywords:** concussion, neuroinflammation, pediatric, vasculature

## Abstract

Traumatic brain injury (TBI) disrupts the blood–brain barrier (BBB), which may exacerbate neuroinflammation post-injury. Few translational studies have examined BBB dysfunction and subsequent neuroinflammation post-TBI in juveniles. We hypothesized that BBB dysfunction positively predicts microglial activation and that vulnerability to BBB dysfunction and associated neuroinflammation are dependent on age at injury. Post-natal day (PND)17 and PND35 rats (*n* = 56) received midline fluid percussion injury or sham surgery, and immunoglobulin-G (IgG) stain was quantified as a marker of extravasated blood in the brain and BBB dysfunction. We investigated BBB dysfunction and the microglial response in the hippocampus, hypothalamus, and motor cortex relative to age at injury and days post-injury (DPI; 1, 7, and 25). We measured the morphologies of ionized calcium-binding adaptor molecule 1–labeled microglia using cell body area and perimeter, microglial branch number and length, end-points/microglial cell, and number of microglia. Data were analyzed using generalized hierarchical models. In PND17 rats, TBI increased levels of IgG compared to shams. Independent of age at injury, IgG in TBI rats was higher at 1 and 7 DPI, but resolved by 25 DPI. TBI activated microglia (more cells and fewer end-points) in PND35 rats compared to respective shams. Independent of age at injury, TBI induced morphological changes indicative of microglial activation, which resolved by 25 DPI. TBI rats had fewer cells and end-points per cell at 1 and 7 DPI than 25 DPI. Independent of TBI, PND17 rats had larger, more activated microglia than PND35 rats; PND17 TBI rats had larger cell body areas and perimeters than PND35 TBI rats. Importantly, we found support in both ages that IgG quantification predicted microglial activation after TBI. The number of microglia increased with increasing IgG, whereas branch length decreased with increasing IgG, which together indicate microglial activation. Our results suggest that stabilization of the BBB after pediatric TBI may be an important therapeutic strategy to limit neuroinflammation and promote recovery.

## Introduction

The incidence of traumatic brain injury (TBI) peaks in toddlers (0–4 years of age) and adolescents (15–18 years of age), which suffer a high frequency of TBIs because of participation in contact sports as well as from domestic violence, car accidents, and falls.^1^ Both early childhood and adolescence are critical time periods for brain development,^1,2^ and little is known about the effect of TBI on the brain during these developmental stages.

The vasculature of the brain, commonly referred to as the blood–brain barrier (BBB), regulates the free flow of blood and its constituents into and out of the brain.^3^ Astrocytes, pericytes, microglia, smooth muscle cells, perivascular microglia, and neurons work cohesively to regulate translocation through the BBB.^3^ TBI often causes tearing of blood vessels and extracellular matrix, which leads to contusion and hemorrhage.^4^ Damage to vascular structures can also alter their long-term function, thereby decreasing cerebral blood flow,^4,5^ cause poorer patient outcomes,^6^ increase inflammation, and heighten the risk for secondary injury.^7^ The BBB plays a critical role in development by facilitating the transport of hormones and trophic factors into the brain^3,8^; however, few translational studies have examined BBB dysfunction and subsequent neuroinflammation after TBI.

Microglia play a vital role in BBB function,^9,10^ and these innate immune cells also respond to damage and infection. Once microglia have detected an inflammatory stimulus, they rapidly respond to protect the brain from further damage.^11^ A hallmark feature of microglial activation after TBI is a continuum of morphological shifts, which, collectively, are commonly used as a marker of inflammation and pathology.^11–13^ In this study, an activated microglial morphology included the following measures: increased cell soma size; decreased branching; and increased cell number in a region of interest. Acutely, the microglial response is beneficial and clears the dead cells, chemical messengers, and extravasated blood caused by TBI.^14^ However, when microglial activation progresses chronically, inflammatory mediators remain elevated, further increasing the inflammatory response and permeability of the BBB.^15^ Consequently, a self-perpetuating proinflammatory loop is created that causes additional damage to the brain.

A spatiotemporal relationship exists between BBB disruption and microglial activation.^16,17^ Here, we investigated the morphological changes of microglia, and quantified immunoglobulin-G (IgG) as a marker of extravasated blood in the brain and overall BBB dysfunction, after midline fluid percussion injury (mFPI) in post-natal day (PND)17 (equivalent to early childhood in humans) and PND35 (equivalent to adolescence in humans) rats. We also investigated whether the amount of extravasated blood in the brain (IgG score) predicted the extent of microglial activation. We hypothesized that TBI would increase microglial activation and extravasated blood in the brain, in both PND17 and PND35 rats, but to a greater extent in PND17 rats.

## Methods

### Study design

**FIG. 1.**
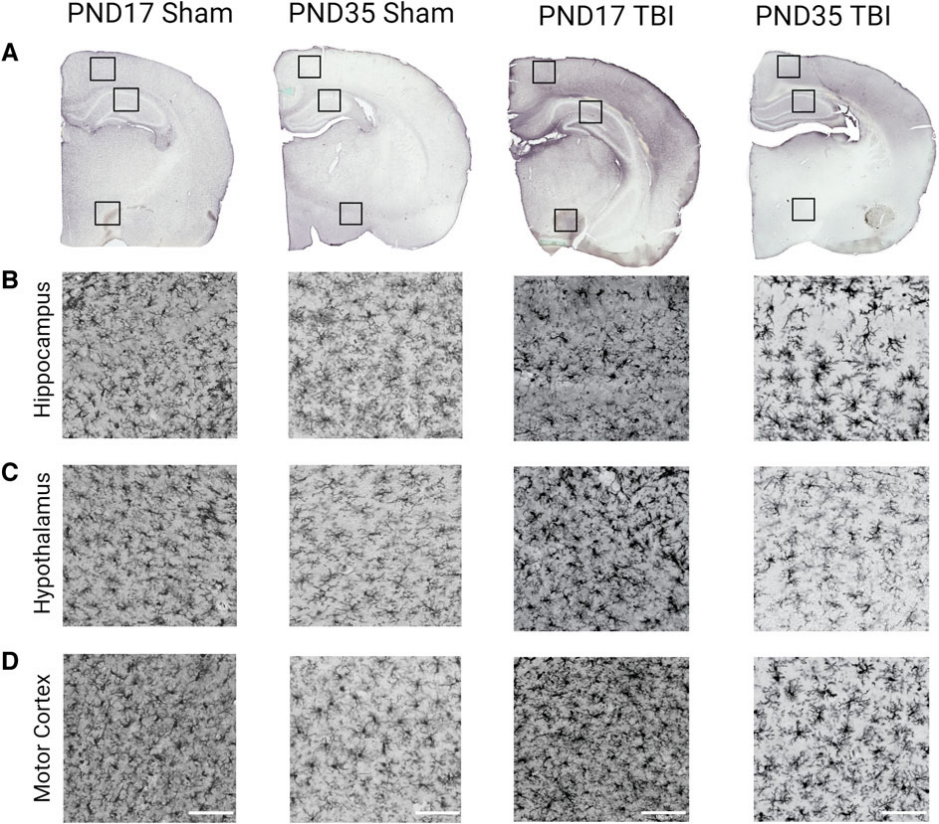
IgG and Iba1 staining in PND17 and PND35 rats at 1 DPI. (**A**) IgG staining in PND17 sham, PND35 sham, PND17 TBI, and PND35 TBI 1 day after mFPI or control sham surgery. Iba1 staining in the (**B**) hippocampus, (**C**) hypothalamus, and (**D**) motor cortex of PND17 sham, PND35 sham, PND17 TBI, and PND35 TBI 1 day after mFPI or control sham surgery. Scale bar = 100 μm. DPI, days post-injury; Iba1, ionized calcium-binding adaptor molecule 1; IgG, immunoglobulin-G; mFPI, midline fluid percussion injury; PND, post-natal day; TBI, traumatic brain injury.

PND17 and PND35 rats were subjected to mFPI or sham surgery, and brains were collected at pre-determined end-points (1, 7, and 25 days post-injury [DPI]). We investigated vascular permeability and BBB compromise using IgG staining to detect extravasated blood in brain tissue^18^ and microglial activation in the hippocampus, hypothalamus, and motor cortex with immunohistochemistry (Fig. 1). We also investigated whether extravasated blood in the brain predicted microglial activation after mFPI relative to age at injury and time post-injury.

### Rigor

Experiments followed the National Institutes of Health and Institutional Animal Care and Use Committee (IACUC) guidelines. The Animal Research: Reporting in vivo Experiments (ARRIVE) guidelines were also followed. For data analysis, a total of 56 rats were used. Exclusion criteria were if rats lost >20% of their body weight or had a breached dura during surgery; however, no rats in the study met these criteria. Pre-determined inclusion criteria included a righting reflex time >120 sec, loss of <20% body weight, and an intact dura. PND17 rats had an average righting reflex time of 204 ± 94 sec with an average apnea time of 10 ± 8 sec. PND35 rats had an average righting reflex time of 409 ± 80 sec with an average apnea time of 6 ± 8 sec. All samples and files were labeled with code names by an investigator not associated with the current study to ensure that all experiments were conducted under blinded conditions. Group sample sizes were: PND17 sham 1 DPI *n* = 4; PND17 sham 7 DPI *n* = 4; PND17 sham 25 DPI *n* = 4; PND17 TBI 1 DPI *n* = 5; PND17 TBI 7 DPI *n* = 6; PND17 TBI 25 DPI *n* = 5; PND35 sham 1 DPI *n* = 6; PND35 sham 7 DPI *n* = 4; PND35 sham 25 DPI *n* = 4; PND35 TBI 1 DPI *n* = 4; PND35 TBI 7 DPI *n* = 5; and PND35 TBI 25 DPI *n* = 5.

### Animals

Male Sprague-Dawley rats (Envigo, Indianapolis, IN) were housed in a 12-h light/12-h dark cycle at 23°C ± 2°C with food and water available *ad libitum* according to the Association for Assessment and Accreditation of Laboratory Animal Care International guidelines. Male rats were used in this pilot study primarily because of sex differences in hormones during puberty, which could cause differential responses to injury between sexes of juveniles.^19^ Additional studies that include female rats are ongoing. Rats were acclimated from shipping for 1 week before experiments. Rats injured at PND17 arrived at PND10 and acclimated with the dam. Rats injured at PND35 arrived at PND28, which was post-weaning, so litter information was not available from the vendor. Rats were randomly assigned to a treatment group (mFPI or sham surgery), using block randomization to ensure balance of sample sizes across treatments and time points. After surgery, post-operative care by physical examination took place. PND17 rats were returned to their dam after surgery and mFPI until tissue collection (1 or 7 DPI) or until they were weaned at PND24. Animal care and experiments were approved by an IACUC at the University of Arizona (protocol 13–460).

### Midline fluid percussion injury

For surgery, all rats were administered 5% isoflurane in 100% oxygen for 5 min and then secured in a stereotaxic frame. Anesthetization was maintained with continuous isoflurane delivery at 2.5% by nosecone. A midline incision was made, and a craniectomy (outer diameter 3 mm in PND17 rats and 4 mm in PND35 rats) was trephined midway between bregma and lambda, as previously published.^20,21^ Craniectomy size was adjusted based on our previously published calculations to account for the smaller skull size in PND17 rats compared to PND35 rats.^20^ The skull flap was removed with care not to disrupt the dura or superior sagittal sinus underlying the craniectomy site. An injury hub, prepared from the female portion of a Luer-Loc needle hub, was fixed over the craniectomy using cyanoacrylate gel and methyl-methacrylate (Hygenic Corp., Akron, OH). Post-surgery, rats were placed on a heating pad and monitored until ambulatory.

Approximately 60–120 min after surgery, rats were subjected to mFPI with methods previously described for PND17 and PND35 rats.^20,21^ Briefly, rats were reanesthetized with 5% isoflurane in 100% oxygen delivered for 3 min. The hub assembly was filled with saline, and PND35 rats were attached directly to the FPI device (Custom Design and Fabrication, Richmond, VA). PND17 rats were connected to the injury device by an extension tube. When a pedal withdrawal response was detected, the pendulum was released causing a fluid pulse directly onto the intact dura resulting in a mild-to-moderate TBI in all rats (PND17 = 1.5 atmospheres pressure [atm], PND35 = 1.9 atm).^20,21^ Sham rats were connected to the device, but the pendulum was not released.

Hubs were removed immediately after injury or sham injury, and rats were monitored for apnea, righting reflex time (time from the initial impact until the rat spontaneously righted itself from a supine position), and a fencing response.^22^ After rats spontaneously righted, brains were inspected for herniation, hematomas, and integrity of the dura. Sham rats spontaneously righted (∼20 sec) when removed from the device. Rats were briefly reanesthetized, and scalp incisions were cleaned with sterile saline and closed. Rats were placed in a heated recovery cage and monitored until ambulatory. Rat welfare was evaluated and documented daily during post-operative care by physical examination.

### Cryoprotection and tissue sectioning

At pre-determined time points post-injury (1, 7, and 25 days), a lethal dose of Euthasol^®^ was administered intraperitoneally. Rats underwent transcardial perfusion with iced 4% paraformaldehyde (PFA) after flushing with phosphate-buffered saline (PBS). Brains were removed from skulls and drop-fixed in 4% PFA for 24 h. Brains were incubated in 15% and then 30% sucrose for 24 h each. Brains were then hemisected, and the left hemisphere from each rat was frozen in optimal cutting temperature compound in groups of 6–9, using the Megabrain technique where multiple brains were mounted and cut together as previously published.^23^ Megabrains were cryosectioned coronally at 40 μm and mounted on slides. Slides were stored at −80°C until immunohistochemistry.

### Immunoglobulin-G immunohistochemistry and analysis

Slides were removed from the −80°C freezer and baked for 3 h at 60°C. Slides were then washed in PBS (3 × 2 min), and a PAP pen was applied to the perimeter of the slide. Blocking solution (10% normal goat serum [NGS], 1% Triton X-100 in PBS) was applied to each slide and left to incubate for 120 min. Blocking solution was removed, and antibody solution (biotinylated antirat IgG [H + L]; catalog no. BA-9401 at 1:250 concentration in 1% NGS and PBS; Vector Laboratories, Inc., Newark, CA) was applied to each slide and left to incubate at room temperature for 60 min. Slides were washed in PBS. Endogenous peroxidases were blocked by placing slides in 200 mL of PBS +8 mL of H_2_O_2_ for 30 min.

After washing in PBS, avidin-biotin complex (ABC) solution (catalog no. 32020; ThermoFisherScientific, Waltham, MA) was applied and left to incubate for 30 min. Slides were washed in PBS, and 3,3′-diaminobenzidine (DAB; catalog no. ab64259; Abcam, Cambridge, MA) solution (four drops of DAB, two drops of H_2_O_2_, and two drops of nickel per 5 mL of PBS) was applied with an incubation time of 3 min. Slides were placed in water for 5 min and then successively placed in 70% and 90% ethanol, methyl green (4 g of methyl green Sigma-Aldrich catalog no. 323829, 25 g in 200 mL of methanol; Sigma-Aldrich, St. Louis, MO), and 100% ethanol. Slides were placed in CitroSolve, and cover-slips were applied using DPX mounting medium. Two IgG-stained slides from between bregma and lambda were analyzed under the microscope at successive magnifications of 5–40 × to determine the presence or absence of bleeding in the following seven areas: peri-injury cortex; primary somatosensory barrel field cortex; above the cornu ammonis (CA) 3 region/beside the CA1 region/below the dentate gyrus of the hippocampus; hypothalamus; or other.

These data were analyzed using a binary score to determine the presence or absence of bleeding in the given areas and summed to give a cumulative score (minimum score 0, maximum score 7). A total of 80 μm was scored for each rat and averaged by investigators blinded to experimental conditions.

### Ionized calcium-binding adaptor molecule 1 immunohistochemistry

Tissue was baked for 3 h and then rehydrated in PBS. Slides were placed in sodium citrate buffer (pH 6.0) and microwaved at power 7 (Whirlpool 1100 watts microwave; Whirlpool, Benton Harbor, MI) for 3 min and power 3 for 10 min, with a previous cooling time of 90 min. Slides were then washed in PBS, and a PAP pen was applied to the perimeter of the slide. Slides were incubated in blocking solution (4% normal horse serum [NHS], 0.1% Triton-100 in PBS) for 60 min. Slides were incubated in primary antibody solution (rabbit anti-Iba1 [ionized calcium-binding adaptor molecule 1]; WAKO catalog no. 019-919741 at 1:1000 concentration in 1% NHS, 0.1% Triton-100 in PBS; Wako Pure Chemical Industries, Ltd, Richmond, VA) overnight at 4°C. Slides were then washed in PBS +0.1% Tween-20. Secondary antibody solution (biotinylated horse antirabbit IgG [H+L]; vector BA-1100 at 1:250 concentration in 4% NHS and 0.4% Triton-100 in PBS) was applied for 60 min at room temperature. Tissue was then washed in PBS, and endogenous peroxidases were blocked by placing slides in 200 mL of PBS +8 mL of H_2_O_2_ for 30 min. After washing in PBS and 0.1% Tween-20, ABC solution (Vectastain ABC kit PK-6100) was applied for 30 min.

Slides were washed in PBS +0.1% Tween-20, and DAB solution (Vector DAB peroxidase substrate kit SK-4100) was applied to the slide for 10 min and immediately placed in water for 10 min. Slides were dehydrated by placing in successive concentrations (70%, 90%, and 100% × 2) of ethanol. Tissue was placed in CitroSolve, and slides were cover-slipped using DPX mounting medium. Z-stack images of Iba1-stained slides were taken using the Leica Aperio slide scanner with Aperio ImageScope software (Leica Biosystems, Deer Park, IL).

### Microglia morphology analysis

For each rat, three slices per animal were imaged and analyzed using ImageJ and the skeletal analysis plugin (NIH, Bethesda, MD).^24^ Regions of interest included the hypothalamus, motor cortex, and hippocampus. Consistent steps were used to process images before converting to binary. Microglial cell somas were counted and three microglial cells per image were isolated, and areas and perimeters were measured. Images were converted to binary and skeletonized, and the analyze skeleton plugin was applied to measure the average number of branches, branch length, and number of end-points per microglial cell captured in 40 × photomicrographs.^13,21,25^ Skeleton analysis measurements captured data for all microglia that appeared in the photomicrographs. To ensure authenticity to the original image, a skeletonized overlay was created to ensure accuracy and consistency to each individual image. Total microglial count, process length, and end-points were recorded and averaged per number of cells in each region of interest. However, these data points were not averaged across images for an animal. Data from a single image were retained as an individual data point, based on our previously published methods.^11^

### Statistical analyses

The study design and data-generating processes were hierarchical, so we conducted all data analysis using generalized linear mixed-effects models that we fit in the R statistical computing environment using the package glmmTMB.^26,27^ We fit two models to each outcome/response variable of interest: one with a treatment × DPI fixed-effects interaction and one with a treatment × age-at-injury fixed-effects interaction. Although IgG values were ordinal, subsequent to exploratory analyses, we chose to treat IgG as arising from a zero-truncated approximately normal distribution, specifying a Gaussian error distribution in all IgG outcome models.^28^ All IgG outcome models included random intercepts for cohort to account for potential variation that may have been induced by cohorts of participants being subjected to injuries on different days.^11,29^ Microglial morphology data were subset by region, and region-specific models were fit. All microglial morphology outcome models were treated as integer count data; the number of microglial cells outcome satisfied the Poisson assumption of equal mean and variance, so a Poisson error distribution was used, whereas the number of branches, branch lengths, number of end-points, cell body area, and cell body perimeter exhibited overdispersion for which negative-binomial error distributions were used.^11,30^

All microglial measures outcome models included random intercepts for individual rats to accommodate the clustering (correlation) of multiple data points being obtained for each rat, and those animals were implicitly nested within random intercepts for litter, which accounted for potential clustering caused by rats originating from the same litter for PND17 rats; PND35 rats were all assigned to the same litter identification because their true litters were unknown. For the separate IgG predictive models, we subset the data to exclude 25 DPI because the presence of IgG after TBI resolved by 25 DPI. In each predictive model, we specified an IgG × injury fixed-effects interaction.

Inference from all models was made based on a combination of predicted conditional effects point estimates and their 95% confidence intervals, *p* values for between-group comparisons from *post hoc* Tukey-adjusted multiple comparisons tests, and effect sizes of group differences (*d* for categorical comparisons, *r* for continuous comparisons), all of which we produced using the R packages ggeffects and emmeans.^31^ Between-group differences were considered statistically significant if *p* < 0.05, whereas between-group differences were considered biologically significant (important) if effect sizes were *d* > 0.2; thus, we placed more inferential weight on effect sizes than *p* values because effect sizes are invariant to sample sizes.^32,33^ Distributions of the raw data are provided as background violins on results figures of the predicted conditional effects.

## Results

### Diffuse traumatic brain injury increased immunoglobulin-G scores in post-natal day 17 rats but not in post-natal day 35 rats

**FIG. 2.**
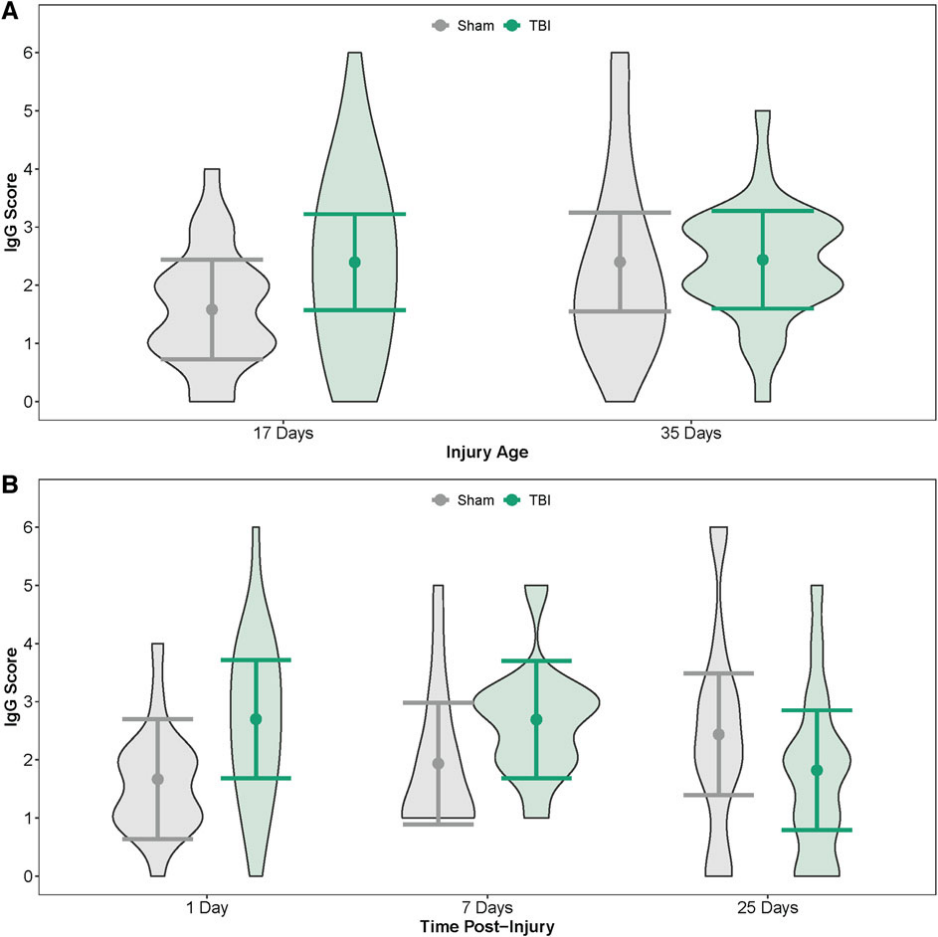
Amount of IgG in the brain by injury age and time post-injury. (**A**) Predicted IgG scores of the amount of IgG in the brain at PND17 and PND35. (**B**) Predicted IgG scores of the amount of IgG in the brain across time post-injury (injury ages combined). Results presented as the estimated conditional effects point estimates (dots) and their corresponding 95% confidence intervals (error bars) from generalized linear mixed-effects models. Distributions of the raw data are represented by the background violins. IgG, immunoglobulin-G; PND, post-natal day; TBI, traumatic brain injury.

IgG staining was greater in PND17 TBI rats than in age-matched shams and greater in PND35 shams than PND17 shams (Fig. 2A; Table 1). In contrast, IgG staining did not differ between PND35 TBI rats and PND35 shams, nor did IgG differ between PND17 TBI rats and PND35 TBI rats. When age was disregarded (i.e., PND17 and PND35 rats grouped together), TBI rats had greater IgG staining than shams at both 1 and 7 DPI, whereas TBI rats had less IgG than shams at 25 DPI (Fig. 2B; Table 2).

**Table 1.** Estimated *p* Values and Effect Sizes for Comparisons of IgG Scores Among Injury × Age Group Combinations from a Generalized Linear Mixed Model With Gaussian Error Distribution

Comparison	*p *value	Effect size
Sham PND17 vs. TBI PND17	0.05	**0.71**
Sham PND17 vs. sham PND35	0.55	**0.71**
TBI PND17 vs. TBI PND35	0.99	0.04
Sham PND35 vs. TBI PND35	0.99	0.03

Statistical significance was achieved if *p* < 0.05, whereas biological significance was achieved if effect size >0.20. Bold values denote biologically important effects.

IgG, immunoglobulin-G; PND, post-natal day; TBI, traumatic brain injury.

**Table 2.** Estimated *p* Values and Effect Sizes for Comparisons of IgG Scores Among Injury × Time Post-Injury Group Combinations from a Generalized Linear Mixed Model With Gaussian Error Distribution

Comparison	*p *value	Effect size
Sham 1 DPI vs. TBI 1 DPI	0.06	**0.93**
Sham 1 DPI vs. sham 7 DPI	0.99	**0.24**
Sham 1 DPI vs. sham 25 DPI	0.89	**0.69**
TBI 1 DPI vs. TBI 7 DPI	0.99	0.01
TBI 1 DPI vs. TBI 25 DPI	0.82	**0.79**
Sham 7 DPI vs. TBI 7 DPI	0.32	**0.68**
Sham 7 DPI vs. sham 25 DPI	0.97	**0.45**
TBI 7 DPI vs. TBI 25 DPI	0.83	**0.78**
Sham 25 TBI vs. TBI 25 DPI	0.59	**0.55**

Statistical significance was achieved if *p* < 0.05, whereas biological significance was achieved if effect size >0.20. Bold values denote biologically important effects.

IgG, immunoglobulin-G; DPI, days post-injury; TBI, traumatic brain injury.

### In the hippocampus, post-natal day 17 shams had more microglial cells than post-natal day 35 shams

**FIG. 3.**
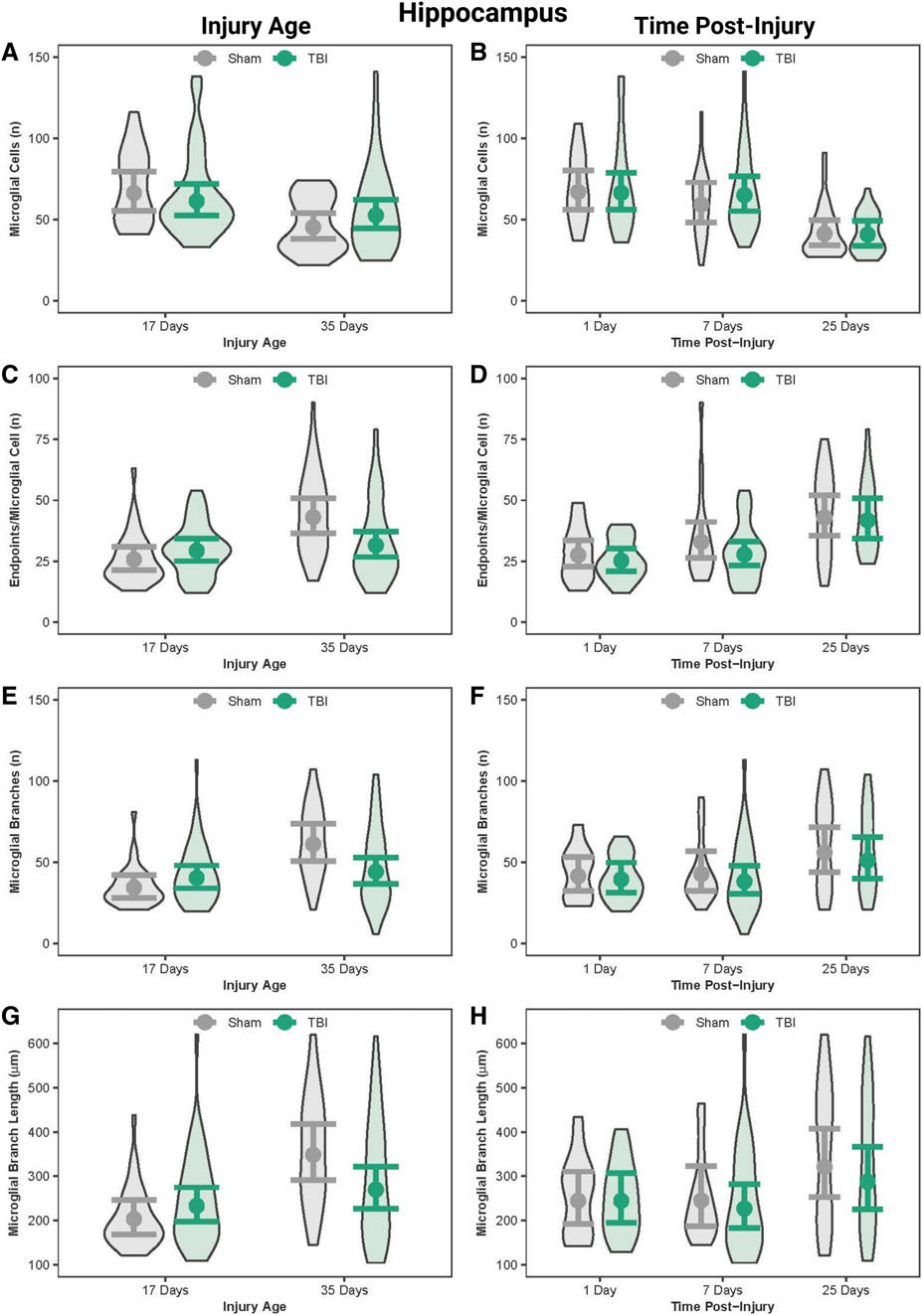
Microglial morphology in the hippocampus by injury age and time post-injury. (**A**) Predicted number of microglial cells across age at injury and (**B**) time post-injury. (**C**) Predicted number of end-points per microglia across age at injury and (**D**) time post-injury. (**E**) Predicted number of processes per microglia across age at injury and (**F**) time post-injury. (**G**) Predicted branch length per microglia across age at injury and (**H**) time post-injury. Results presented as the estimated conditional effects point estimates (dots) and their corresponding 95% confidence intervals (error bars) from generalized linear mixed-effects models. Distributions of the raw data are represented by the background violins. TBI, traumatic brain injury.

In the hippocampus, PND17 shams had more microglial cells than PND35 shams; however, the number of microglial cells were similar between PND17 TBI and PND35 TBI (Fig. 3; Table 3). PND17 shams had fewer number of branches and end-points/microglia cells, and shorter branch lengths compared to PND35 shams. PND35 TBI rats had fewer end-points/microglia compared to PND35 shams. Although multiple age effects on microglial morphology were statistically significant, they had small effect sizes.

**Table 3.** Estimated *p* Values and Effect Sizes for Comparisons of Microglia Morphological Outcomes in the Hippocampus Among Injury × Age Group Combinations from Generalized Linear Mixed Models With Negative-Binomial or Poisson Error Distributions

Comparison	No. microglia		No. branches		Branch length		No. end-points	
	*p *value	Effect size	*p *value	Effect size	*p *value	Effect size	*p *value	Effect size
Sham PND17 vs. TBI PND17	0.92	0.08	0.62	0.01	0.72	0.01	0.71	0.01
Sham PND17 vs. sham PND35	0.02	**0.38**	<0.001	0.03	<0.001	0.03	<0.001	0.03
TBI PND17 vs. TBI PND35	0.57	0.15	0.90	0.004	0.63	0.01	0.92	0.004
Sham PND35 vs. TBI PND35	0.62	0.15	0.08	0.01	0.20	0.01	0.05	0.02

Statistical significance was achieved if *p* < 0.05, whereas biological significance was achieved if effect size >0.20. Bold values denote biologically important effects.

PND, post-natal day; TBI, traumatic brain injury.

### Traumatic brain injury transiently activated microglia in the acute post-injury period in the hippocampus

In the hippocampus, shams at both 1 and 7 DPI had substantially more microglial cells than shams at 25 DPI; similarly, TBI rats at both 1 and 7 DPI had substantially more microglial cells than TBI rats at 25 DPI (Fig. 3, Table 4). Microglial end-points increased as a function of time post-injury. When age was disregarded, TBI rats had fewer end-points/microglia at 1 and 7 DPI compared to 25 DPI. Time effects on microglia morphology were statistically significant, but had small effect sizes. These results collectively suggest that TBI-induced microglial activation peaked in the first 7 DPI and resolved by 25 DPI.

**Table 4.** Estimated *p* Values and Effect Sizes for Comparisons of Microglia Morphological Outcomes in the Hippocampus Among Injury × Time Post-Injury Group Combinations from Generalized Linear Mixed Models With Negative-Binomial or Poisson Error Distributions

Comparison	No. microglia		No. branches		Branch length		No. end-points	
	*p *value	Effect size	*p *value	Effect size	*p *value	Effect size	*p *value	Effect size
Sham 1 DPI vs. TBI 1 DPI	0.99	0.01	0.99	0.002	0.99	<0.001	0.98	0.01
Sham 1 DPI vs. sham 7 DPI	0.94	0.12	0.99	0.001	0.99	<0.001	0.85	0.01
Sham 1 DPI vs. sham 25 DPI	0.004	**0.48**	0.54	0.01	0.61	<0.001	0.02	0.02
TBI 1 DPI vs. TBI 7 DPI	0.99	0.02	0.99	0.001	0.99	<0.001	0.98	0.01
TBI 1 DPI vs. TBI 25 DPI	0.003	**0.49**	0.67	0.01	0.92	<0.001	0.004	0.03
Sham 7 DPI vs. TBI 7 DPI	0.97	0.09	0.97	0.01	0.99	<0.001	0.84	0.01
Sham 7 DPI vs. sham 25 DPI	0.12	**0.36**	0.71	0.01	0.69	<0.001	0.48	0.01
TBI 7 DPI vs. TBI 25 DPI	0.004	**0.47**	0.53	0.01	0.71	<0.001	0.03	0.02
Sham 25 TBI vs. TBI 25 DPI	0.99	0.01	0.98	0.004	0.98	<0.001	0.99	0.002

Statistical significance was achieved if *p* < 0.05, whereas biological significance was achieved if effect size >0.20. Bold values denote biologically important effects.

DPI, days post-injury; TBI, traumatic brain injury.

### In the hypothalamus, post-natal day 17 rats had more microglial cells than post-natal day 35 rats

**FIG. 4.**
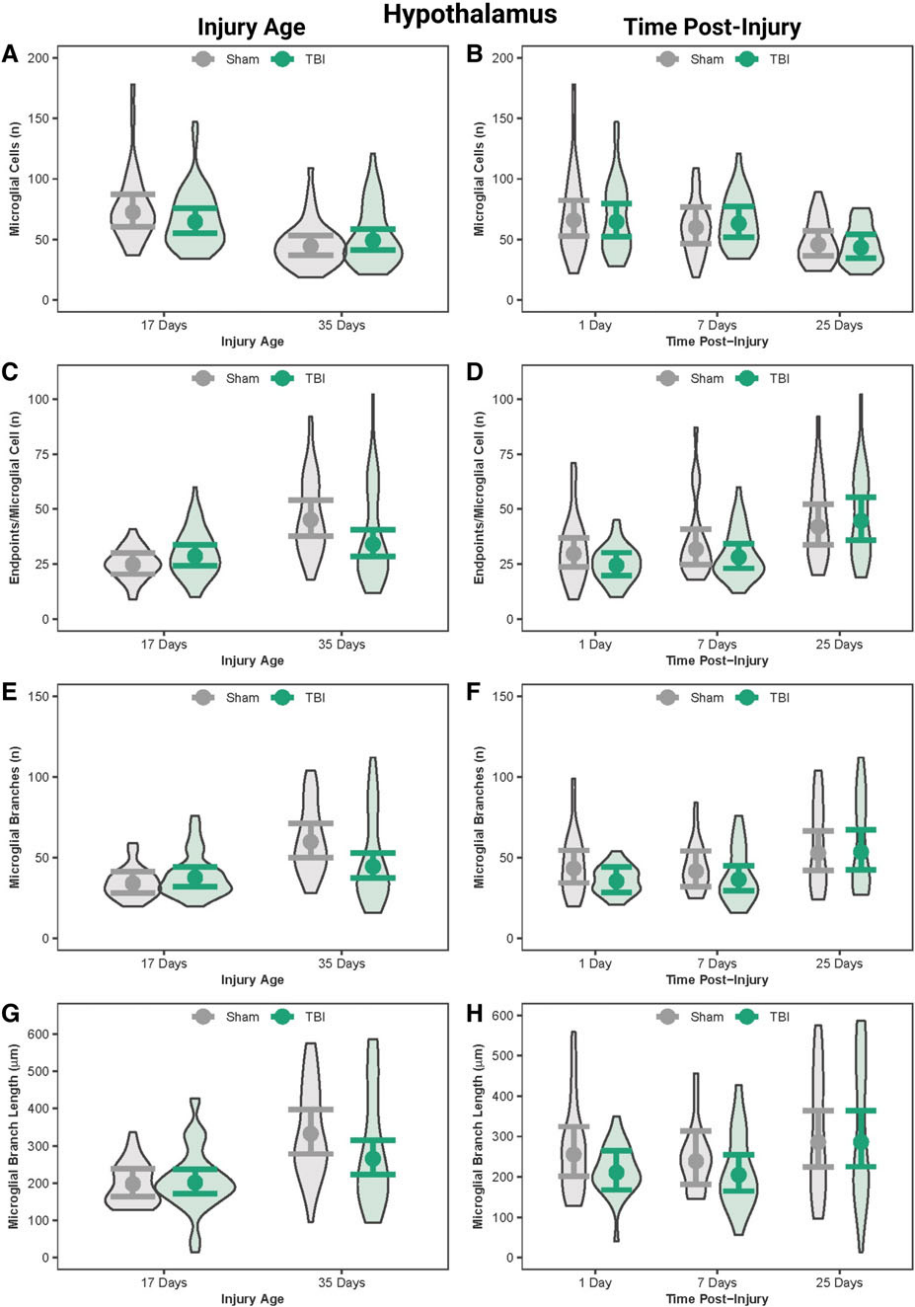
Microglial morphology in the hypothalamus by injury age and time post-injury. (**A**) Predicted number of microglial cells across age at injury and (**B**) time post-injury. (**C**) Predicted number of end-points per microglia across age at injury and (**D**) time post-injury. (**E**) Predicted number of processes per microglia across age at injury and (**F**) time post-injury. (**G**) Predicted branch length per microglia across age at injury and (**H**) time post-injury. Results presented as the estimated conditional effects point estimates (dots) and their corresponding 95% confidence intervals (error bars) from generalized linear mixed-effects models. Distributions of the raw data are represented by the background violins. TBI, traumatic brain injury.

In the hypothalamus, both PND17 sham and TBI rats had more microglial cells than PND35 sham and TBI rats, respectively (Fig. 4; Table 5). PND17 shams had fewer number of branches and end-points/microglia cells and shorter branch lengths compared to PND35 shams. Age effects on microglial morphology were statistically significant, but effect sizes were nominal.

**Table 5.** Estimated *p* Values and Effect Sizes for Comparisons of Microglia Morphological Outcomes in the Hypothalamus Among Injury × Age Group Combinations from Generalized Linear Mixed Models With Negative-Binomial or Poisson Error Distributions

Comparison	No. microglia		No. branches		Branch length		No. end-points	
	*p *value	Effect size	*p *value	Effect size	*p *value	Effect size	*p *value	Effect size
Sham PND17 vs. TBI PND17	0.79	0.12	0.87	0.002	0.99	0.002	0.70	0.01
Sham PND17 vs. sham PND35	0.002	**0.49**	<0.001	0.01	<0.001	0.05	<0.001	0.02
TBI PND17 vs. TBI PND35	0.11	**0.27**	0.51	0.004	0.11	0.02	0.50	0.01
Sham PND35 vs. TBI PND35	0.85	0.10	0.09	0.01	0.29	0.02	0.12	0.01

Statistical significance was achieved if *p* < 0.05, whereas biological significance was achieved if effect size >0.20. Bold values denote biologically important effects.

PND, post-natal day; TBI, traumatic brain injury.

### Microglia were more activated in the hypothalamus at 1 day post-injury and 7 days post-injury than at 25 days post-injury

There were time post-injury effects within sham and within TBI rats; both sham and TBI rats had more microglial cells at 1 DPI and 7 DPI than sham and TBI rats at 25 DPI (Fig. 4; Table 6). Microglial end-points increased as a function of time post-injury. When age was disregarded, TBI rats had fewer end-points/microglial cells at 1 and 7 DPI compared to 25 DPI. Although these differences in TBI rats among time points were statistically significant, the effect sizes were nominal.

**Table 6.** Estimated *p* Values and Effect Sizes for Comparisons of Microglia Morphological Outcomes in the Hypothalamus Among Injury × Time Post-Injury Group Combinations from Generalized Linear Mixed Models With Negative-Binomial or Poisson Error Distributions

Comparison	No. microglia		No. branches		Branch length		No. end-points	
	*p *value	Effect size	*p *value	Effect size	*p *value	Effect size	*p *value	Effect size
Sham 1 DPI vs. TBI 1 DPI	0.99	0.02	0.82	0.01	0.86	<0.001	0.81	0.01
Sham 1 DPI vs. sham 7 DPI	0.97	0.10	0.99	0.001	0.99	<0.001	0.99	0.002
Sham 1 DPI vs. sham 25 DPI	0.21	**0.36**	0.83	0.01	0.99	<0.001	0.25	0.1
TBI 1 DPI vs. TBI 7 DPI	0.99	0.02	0.99	<0.001	0.99	<0.001	0.93	0.01
TBI 1 DPI vs. TBI 25 DPI	0.12	**0.40**	0.12	0.01	0.46	<0.001	0.002	0.02
Sham 7 DPI vs. TBI 7 DPI	0.99	0.06	0.97	0.003	0.95	<0.001	0.98	0.004
Sham 7 DPI vs. sham 25 DPI	0.62	**0.27**	0.76	0.01	0.92	<0.001	0.56	0.01
TBI 7 DPI vs. TBI 25 DPI	0.14	**0.38**	0.16	0.01	0.33	<0.001	0.03	0.02
Sham 25 TBI vs. TBI 25 DPI	0.99	0.05	0.99	<0.001	0.99	<0.001	0.99	0.002

Statistical significance was achieved if *p* < 0.05, whereas biological significance was achieved if effect size >0.20. Bold values denote biologically important effects.

DPI, days post-injury; TBI, traumatic brain injury.

### In the motor cortex, post-natal day 17 rats had more microglial cells than post-natal day 35 rats

**FIG. 5.**
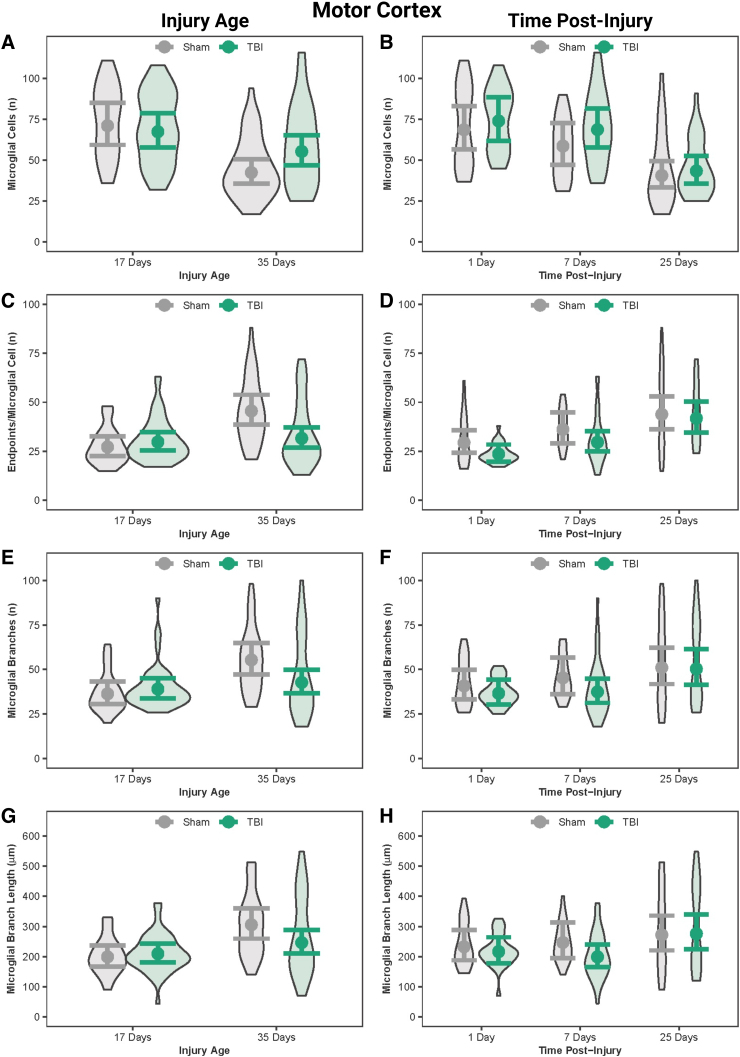
Microglial morphology in the motor cortex by injury age and time post-injury. (**A**) Predicted number of microglial cells across age at injury and (**B**) time post-injury. (**C**) Predicted number of end-points per microglia across age at injury and (**D**) time post-injury. (**E**) Predicted number of processes per microglia across age at injury and (**F**) time post-injury. (**G**) Predicted branch length per microglia across age at injury and (**H**) time post-injury. Results presented as the estimated conditional effects point estimates (dots) and their corresponding 95% confidence intervals (error bars) from generalized linear mixed-effects models. Distributions of the raw data are represented by the background violins. TBI, traumatic brain injury.

In the motor cortex, PND35 TBI rats had substantially more microglial cells than PND35 sham rats (Fig. 5; Table 7). Additionally, PND17 sham and TBI rats had more microglial cells than PND35 sham and TBI rats, respectively. PND17 shams had fewer number of branches and end-points/microglia cells and shorter branch lengths compared to PND35 shams. PND35 TBI rats had fewer end-points/microglial cells compared to PND35 shams. Although statistically significant differences existed in number of microglial branches, branch length, and number of end-points/microglial cells between PND17 sham and PND35 sham rats, as well as between PND35 sham and PND35 TBI rats, the effect sizes for those differences were nominal.

**Table 7.** Estimated *p* Values and Effect Sizes for Comparisons of Microglia Morphological Outcomes in the Motor Cortex Among Injury × Age Group Combinations from Generalized Linear Mixed Models With Negative-Binomial or Poisson Error Distributions

Comparison	No. microglia		No. branches		Branch length		No. end-points	
	*p *value	Effect size	*p *value	Effect size	*p *value	Effect size	*p *value	Effect size
Sham PND17 vs. TBI PND17	0.97	0.05	0.93	0.001	0.97	0.003	0.88	0.003
Sham PND17 vs. sham PND35	<0.001	**0.52**	0.003	0.01	0.003	0.02	<0.001	0.01
TBI PND17 vs. TBI PND35	0.32	**0.20**	0.84	0.002	0.46	0.01	0.95	0.002
Sham PND35 vs. TBI PND35	0.14	**0.27**	0.11	0.01	0.25	0.01	0.01	0.01

Statistical significance was achieved if *p* < 0.05, whereas biological significance was achieved if effect size >0.20. Bold values denote biologically important effects.

PND, post-natal day; TBI, traumatic brain injury.

### Microglia were more activated in the motor cortex at 1 day post-injury and 7 days post-injury than at 25 days post-injury

In the motor cortex, sham and TBI rats at both 1 DPI and 7 DPI had more microglial cells than sham and TBI rats at 25 DPI (Fig. 5; Table 8). When age was disregarded, TBI rats had fewer microglia, and fewer end-points/microglial cells at 1 and 7 DPI compared to 25 DPI; however, the effect sizes were small.

**Table 8.** Estimated *p* Values and Effect Sizes for Comparisons of Microglia Morphological Outcomes in the Motor Cortex Among Injury × Time Post-Injury Group Combinations from Generalized Linear Mixed Models With Negative-Binomial or Poisson Error Distributions

Comparison	No. microglia		No. branches		Branch length		No. end-points	
	*p *value	Effect size	*p *value	Effect size	*p *value	Effect size	*p *value	Effect size
Sham 1 DPI vs. TBI 1 DPI	0.99	0.08	0.97	0.002	0.99	0.004	0.61	0.01
Sham 1 DPI vs. sham 7 DPI	0.89	0.16	0.99	0.002	0.99	0.003	0.74	0.01
Sham 1 DPI vs. sham 25 DPI	0.003	**0.52**	0.63	0.004	0.90	0.01	0.05	0.01
TBI 1 DPI vs. TBI 7 DPI	0.99	0.07	0.99	<0.001	0.99	0.01	0.50	0.01
TBI 1 DPI vs. TBI 25 DPI	0.002	**0.53**	0.21	0.01	0.57	0.01	<0.001	0.02
Sham 7 DPI vs. TBI 7 DPI	0.87	0.15	0.79	0.004	0.73	0.01	0.72	0.01
Sham 7 DPI vs. sham 25 DPI	0.14	**0.37**	0.96	0.002	0.98	0.01	0.78	0.01
TBI 7 DPI vs. TBI 25 DPI	0.01	**0.46**	0.26	0.01	0.21	0.02	0.10	0.01
Sham 25 TBI vs. TBI 25 DPI	0.99	0.07	0.99	<0.001	0.99	<0.001	0.99	0.001

Statistical significance was achieved if *p* < 0.05, whereas biological significance was achieved if effect size >0.20. Bold values denote biologically important effects.

DPI, days post-injury; TBI, traumatic brain injury.

### Diffuse traumatic brain injury increased microglial cell body size immediately after injury

**FIG. 6.**
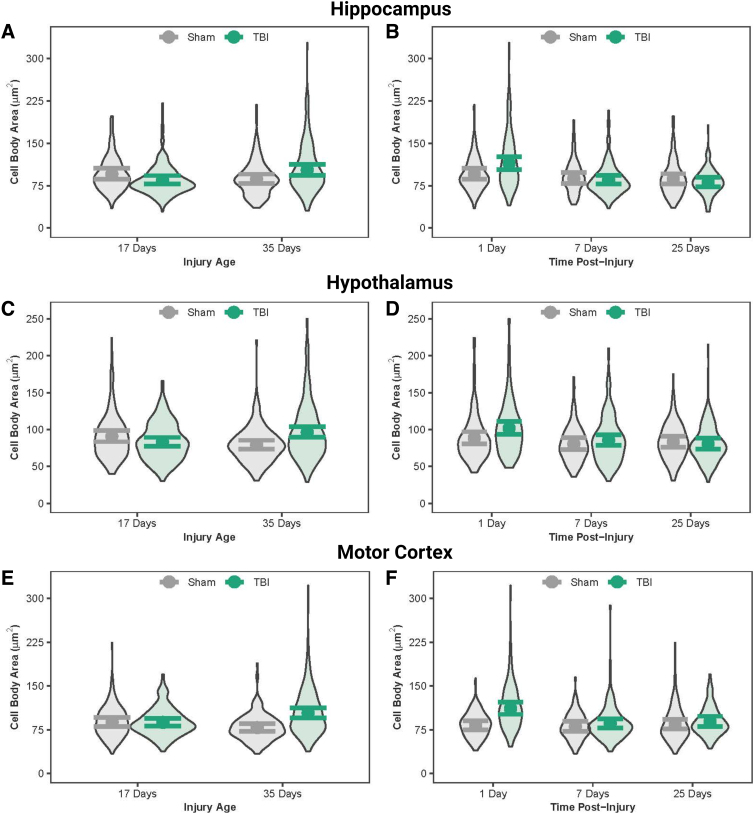
Microglial cell body area in the hippocampus, hypothalamus, and motor cortex. (**A**) Predicted microglial cell body area across age at injury and (**B**) time post-injury in the hippocampus. (**C**) Predicted microglial cell body area across age at injury and (**D**) time post-injury in the hypothalamus. (**E**) Predicted microglial cell body area across age at injury in the motor cortex and (**F**) across time post-injury in the motor cortex. Results presented as the estimated conditional effects point estimates (dots) and their corresponding 95% confidence intervals (error bars) from generalized linear mixed-effects models. Distributions of the raw data are represented by the background violins. TBI, traumatic brain injury.

Cell body area and perimeter were quantified as a measure of microglial activation and cell body complexity. Microglial cell body areas were similar between PND17 sham and TBI rats in all three regions and between PND35 sham and TBI rats in the hippocampus (Fig. 6; Table 9); however, statistically significant differences with nominal effect sizes existed for cell body areas in all three regions between PND17 TBI and PND35 TBI rats, as well as in the hypothalamus and motor cortex between PND35 sham and PND35 TBI rats. PND35 TBI rats had smaller cell body areas in the motor cortex compared to PND35 shams. Immediate time post-injury effects of TBI were strongly supported in all three regions: TBI rats had substantially larger cell body areas than sham rats at 1DPI, whereas cell body areas were similar between TBI and sham rats at 7 DPI and 25 DPI (Fig. 6; Table 10). Additionally, TBI rats had larger cell body areas at 1 DPI than TBI rats at 7 DPI and 25 DPI in all three regions. Differences identical to those described above for cell body areas also existed for cell body perimeters in the three regions (Supplementary Fig. S1; Supplementary Tables S1 and S2).

**Table 9.** Estimated *p* Values and Effect Sizes for Comparisons of Microglial Cell Body Area in Three Brain Regions Among Injury × Age Group Combinations from Generalized Linear Mixed Models With Negative-Binomial Error Distributions

Comparison	Hippocampus		Hypothalamus		Motor cortex	
	*p *value	Effect size	*p *value	Effect size	*p *value	Effect size
Sham PND17 vs. TBI PND17	0.33	0.01	0.41	0.01	0.99	0.001
Sham PND17 vs. sham PND35	0.52	0.01	0.08	0.01	0.29	0.11
TBI PND17 vs. TBI PND35	0.02	0.01	0.03	0.01	0.02	0.17
Sham PND35 vs. TBI PND35	0.06	0.01	0.002	0.02	<0.001	**0.23**

Statistical significance was achieved if *p* < 0.05, whereas biological significance was achieved if effect size >0.20. Bold values denote biologically important effects.

PND, post-natal day; TBI, traumatic brain injury.

**Table 10.** Estimated *p* Values and Effect Sizes for Comparisons of Microglial Cell Body Area in Three Brain Regions Among Injury × Time Post-Injury Group Combinations from Generalized Linear Mixed Models With Negative-Binomial Error Distributions

Comparison	Hippocampus		Hypothalamus		Motor cortex	
	*p *value	Effect size	*p *value	Effect size	*p *value	Effect size
Sham 1 DPI vs. TBI 1 DPI	0.14	**0.20**	0.27	0.14	<0.001	**0.30**
Sham 1 DPI vs. sham 7 DPI	0.87	0.08	0.72	0.10	0.99	0.02
Sham 1 DPI vs. sham 25 DPI	0.77	0.10	0.92	0.06	0.99	0.02
TBI 1 DPI vs. TBI 7 DPI	<0.001	**0.29**	0.05	0.18	<0.001	**0.26**
TBI 1 DPI vs. TBI 25 DPI	<0.001	**0.35**	0.004	**0.23**	0.02	**0.22**
Sham 7 DPI vs. TBI 7 DPI	0.99	0.03	0.94	0.06	0.94	0.06
Sham 7 DPI vs. sham 25 DPI	0.99	0.10	0.99	0.03	0.98	0.05
TBI 7 DPI vs. TBI 25 DPI	0.97	0.05	0.93	0.06	0.99	0.04
Sham 25 TBI vs. TBI 25 DPI	0.93	0.7	0.99	0.03	0.95	0.06

Statistical significance was achieved if *p* < 0.05, whereas biological significance was achieved if effect size >0.20. Bold values denote biologically important effects.

DPI, days post-injury; TBI, traumatic brain injury.

### Immunoglobulin-G score predicted microglial reactivity in the hippocampus, hypothalamus, and motor cortex

**FIG. 7.**
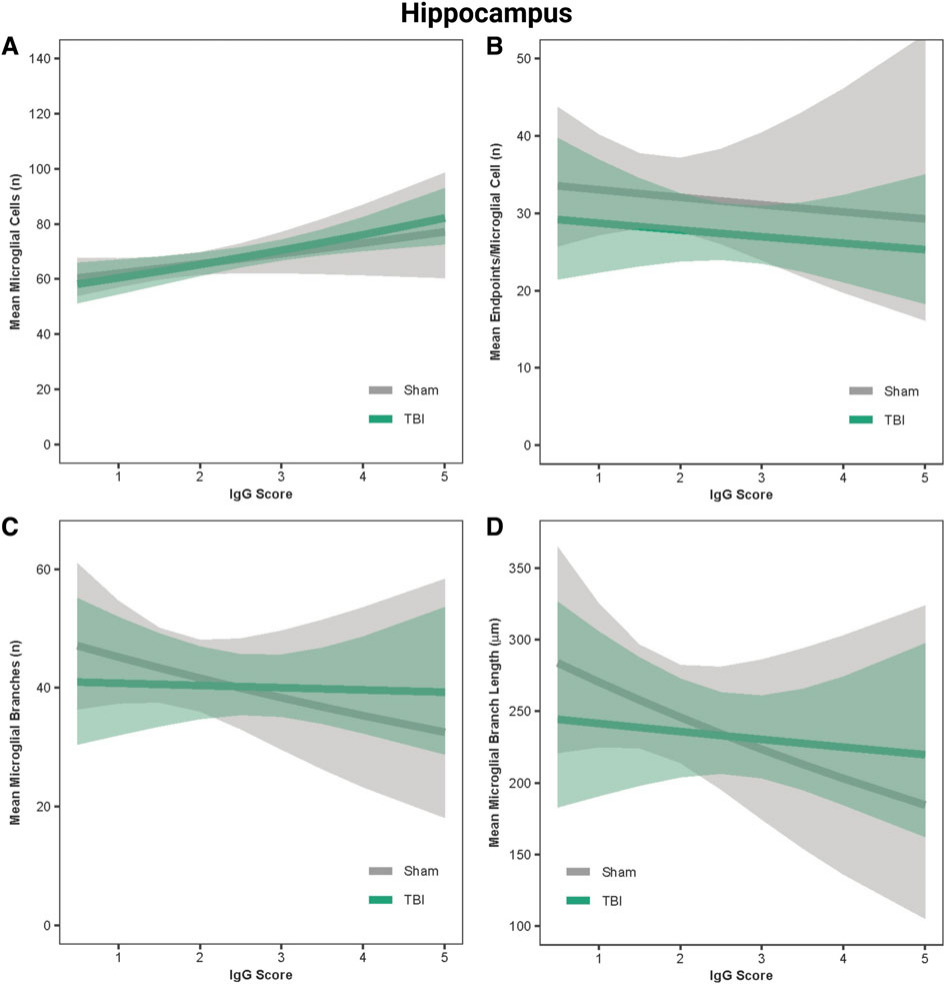
IgG score predicted microglial activation in the hippocampus. (**A**) Predicted relationship between IgG score and the mean number of microglial cells per image. (**B**) Predicted relationship between IgG score and the mean number of end-points. (**C**) Predicted relationship between IgG score and the mean number of microglial branches. (**D**) Predicted relationship between IgG score and the mean microglial branch length per microglial cell. Results presented as the estimated conditional effects point estimates (solid lines) and their corresponding 95% confidence intervals (shaded regions) from generalized linear mixed-effects models. IgG, immunoglobulin-G; TBI, traumatic brain injury.

**FIG. 8.**
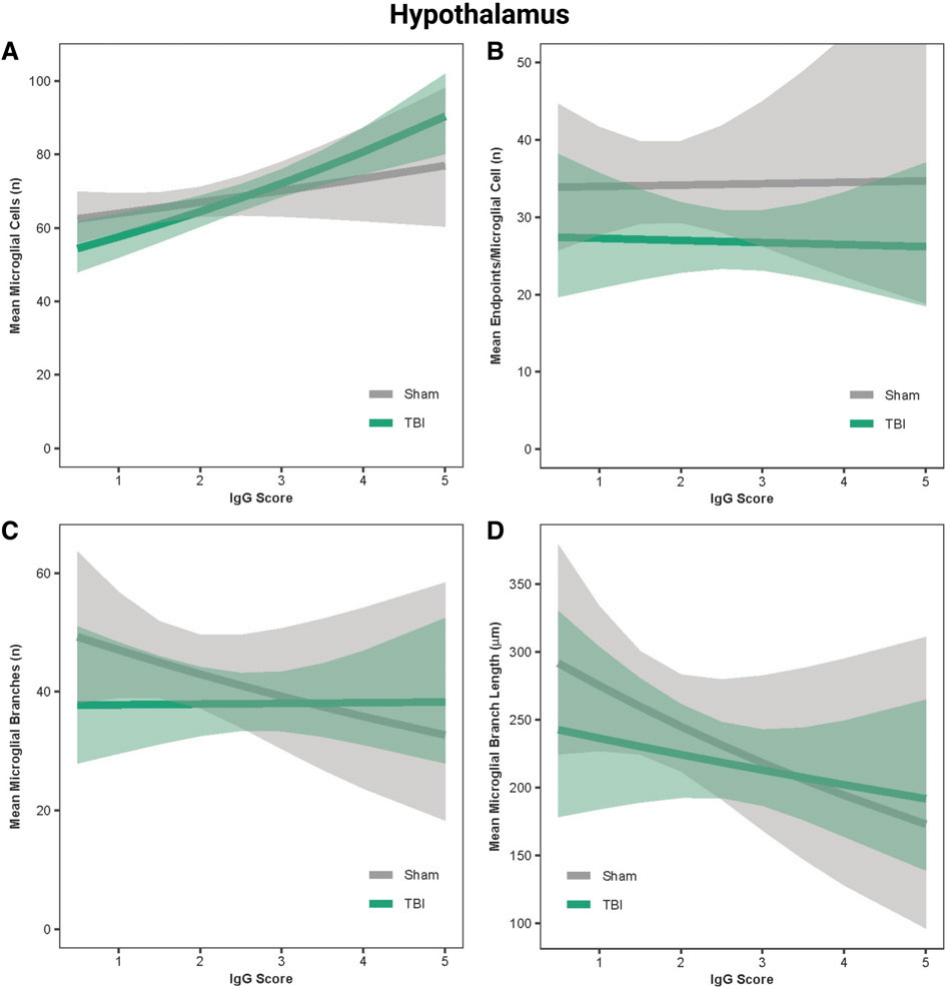
IgG score predicted microglial activation in the hypothalamus. (**A**) Predicted relationship between IgG score and the mean number of microglial cells per image. (**B**) Predicted relationship between IgG score and the mean number of end-points. (**C**) Predicted relationship between IgG score and the mean number of microglial branches. (**D**) Predicted relationship between IgG score and the mean microglial branch length per microglial cell. Results presented as the estimated conditional effects point estimates (solid lines) and their corresponding 95% confidence intervals (shaded regions) from generalized linear mixed-effects models. IgG, immunoglobulin-G; TBI, traumatic brain injury.

**FIG. 9.**
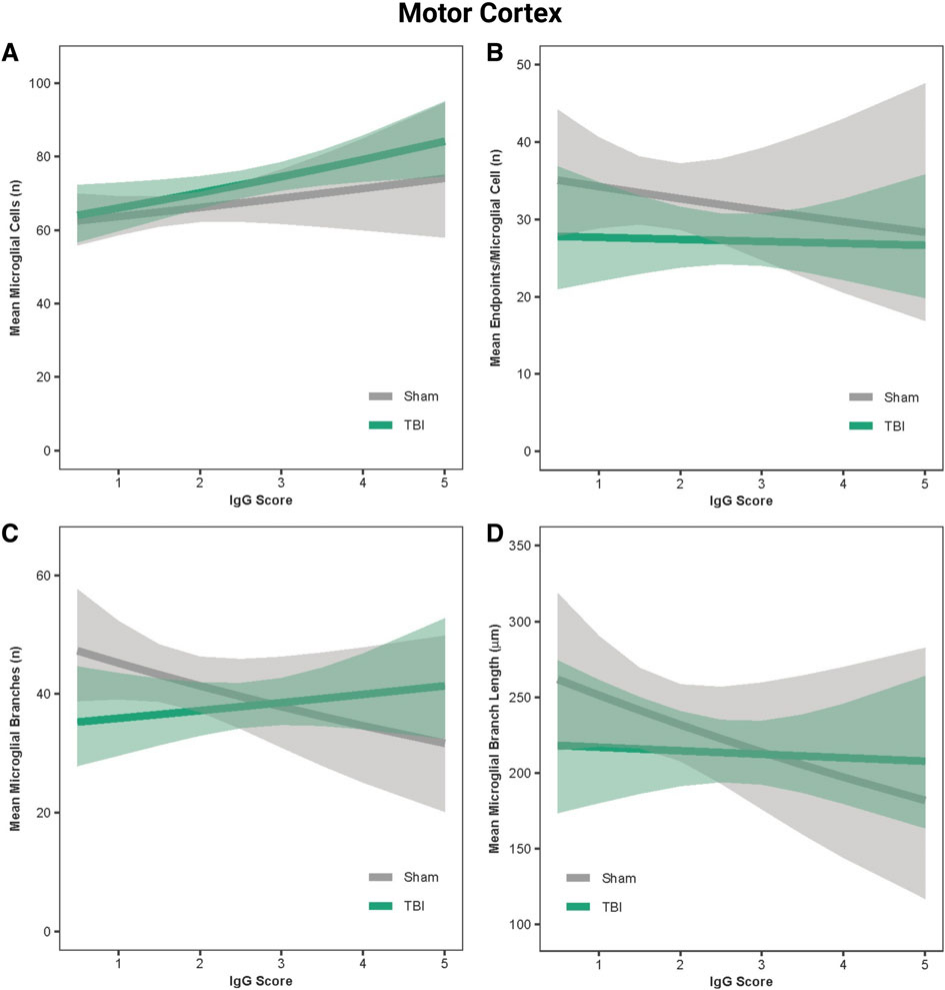
IgG score predicted microglial activation in the motor cortex. (**A**) Predicted relationship between IgG score and the mean number of microglial cells per image. (**B**) Predicted relationship between IgG score and the mean number of end-points. (**C**) Predicted relationship between IgG score and the mean number of microglial branches. (**D**) Predicted relationship between IgG score and the mean microglial branch length per microglial cell. Results presented as the estimated conditional effects point estimates (solid lines) and their corresponding 95% confidence intervals (shaded regions) from generalized linear mixed-effects models. IgG, immunoglobulin-G; TBI, traumatic brain injury.

In the hippocampus, IgG score was a positive predictor of the number of microglial cells in both sham and TBI rats (β = 0.05, *r* = 0.99, *p* < 0.001; Fig. 7A) and a negative predictor of microglial branch lengths and number of branches in sham rats only (β = −0.08 to −0.10, *r* = −0.99, *p* < 0.001; Fig. 7C,D); however, IgG was not predictive of the number of end-points for sham or TBI rats (β = −0.003, *r* = 0.56, *p* = 0.13; Fig. 7B). In the hypothalamus, IgG score was a positive predictor of the number of microglial cells in both sham and TBI rats (β = 0.05, *r* = 0.99, *p* < 0.001; Fig. 8A), a negative predictor of microglial branch lengths in both sham and TBI rats (β = −0.12, *r* = −0.99, *p* < 0.001; Fig. 8D), and a negative predictor of number of branches in sham rats only (β = −0.09, *r* = −0.99, *p* < 0.001; Fig. 8C); however, IgG was not predictive of the number of end-points for sham or TBI rats (β = 0.005, *r* = 0.58, *p* = 0.48; Fig. 8B). In the motor cortex, IgG score was a positive predictor of the number of microglial cells in both sham and TBI rats (β = 0.04, *r* = 0.99, *p* < 0.001; Fig. 9A) and a negative predictor of the number of end-points, microglial branches, and microglial branch lengths in shams only (β = −0.05 to −0.09, *r* = −0.90 to −0.99, *p* < 0.001; Fig. 9B–D).

## Discussion

Breach of the BBB has a spatiotemporal relationship with microglial activation in the brain. We used the amount of IgG staining in the brain and skeletal analysis, a detailed quantitative measure of microglial morphology, and found that the amount of blood in the brain, marked by IgG staining, predicted the extent of TBI-induced microglial activation in rats subjected to TBI at PND17 or PND35. TBI caused significant breach of the BBB in PND17 rats, but this was undetectable in PND35 rats. This finding complements a study that found an increase in IgG in the brain after controlled cortical impact TBI in PND17 rats.^34^ However, TBI in adult rodents has also been shown to cause an increase in IgG after TBI.^35^ To our knowledge, the current study is the first to examine BBB disruption using IgG immunohistochemistry in PND35 rats, although another study used Evans Blue dye to investigate BBB breach in the pituitary gland after a closed TBI in PND35 rodents.^36^ Closed TBI increased the amount of dye in the pituitary gland (suggestive of BBB damage) by 130%, and two impacts increased it further.^37^ The discrepancies between these findings and our own could be attributable to the different techniques used, and given that the pituitary gland is located in the bony sella turcica, this enclosed structure could render the pituitary more vulnerable to BBB breach.

Experimental TBI studies have reported both acute and delayed BBB disruption followed by restoration as evidenced by IgG staining, or intravenously injected tracers including Evans Blue and horseradish peroxidase.^38–41^ When age was disregarded, we found that TBI increased extravasated blood in the brain at 1 and 7 DPI compared to 25 DPI, suggesting a transient permeability of the BBB post-TBI. After lateral fluid percussion injury in adult rats, BBB permeability peaked at 1 h post-injury, with abnormal permeability at 1 DPI that was sustained at 3 DPI.^40^ BBB dysfunction offers the therapeutic opportunity to deliver drugs that are normally excluded from the selectively impermeable brain parenchymal space.^38,42^ Previous experimental TBI studies have focused on adult rodents, so identifying the TBI-induced permeability of the BBB in juvenile rodents addresses a critical gap in the current literature.

Microglial cell soma size is a hallmark feature of microglial activation. We found that TBI increased the cell body area of microglia in the motor cortex, regardless of age at injury, and that cell area increased transiently in all TBI rats. This finding is supported by other studies which have shown that inflammatory microglial markers and activated microglial morphology increased after TBI.^43–45^ We also observed that the microglial cell body area was largest at 1 DPI and decreased over time. Although there are limited data available showing microglial cell soma size at these time points post-TBI in rats, it is well established that microglial activation peaks in the acute time period after injury and decreases thereafter.^14^ Microglia have an ameboid morphology in the peri-injury cortex in the week after injury, but regain ramified branches by 3 weeks post-injury.^46^ In the adult rat, mFPI increased Iba1 staining in the cortex and increased the expression of inflammatory genes at 7 DPI compared to 28 DPI, suggesting that microglial activation peaks acutely after TBI.^47^ Therefore, the results of our study suggest that microglia in the juvenile rodent undergo similar morphological changes after TBI as those shown in adult rats.

Additionally, we found that TBI increased microglial cell body size to a greater extent in PND35 rats compared to PND17 rats. Overall, PND17 shams had more activated microglia than PND35 shams. These findings are supported by our previous data which found that after TBI, microglial cell somas were larger in the retrosplenial and somatosensory cortices of PND35 sham rats compared to PND17 sham rats.^21^ Together, these studies suggest that microglia may be smaller in the uninjured PND17 brain than in the uninjured PND35 brain. Phylogenetic data suggest that the size of microglia is not dependent on brain size^48^; however, there are limited data on whether microglial cell body size changes with age.^21^ We caution that our effect sizes for comparisons of cell body size across age at injury were small despite the differences being statistically significant; therefore, more research is needed to confirm that microglial cell body size increases with age.

PND17 shams had activated microglial morphologies compared to PND35 shams in all regions tested. Given that PND17 rats are in a developmental period where continuous neural circuit remodeling is occurring (post-natal weeks 1–3), it is possible that the increased morphological activation that we observed is a reflection of the microglial role in brain development.^49–51^ Despite the ongoing role of microglia in brain development, microglia reach their full ramification at PND14^52^; consequently, this does not fully explain why microglia at PND17 were more activated than microglia at PND35 in this study. Further research is warranted to characterize microglial morphologies as a function of age during critical developmental stages.

Overall, IgG score predicted microglial activation in the brain across regions. This is supported by findings of other studies that increased BBB permeability upregulates microglial activation.^53–55^ The extent of inflammation and microglial activation in the human brain cannot be measured non-invasively, but hematoma is routinely measured by computed tomography scans after TBI. Deep learning mechanisms have been created to categorize hematomas and inform clinical decisions around craniotomies and other invasive treatments.^56^ Here, we show that the extent of bleeding in juvenile rats predicts microglial activation. TBI patients are routinely screened for hematomas; thus, our findings have potential to inform personalized clinical decisions for post-TBI inflammation treatments. Further, our results provide groundwork for the feasibility of drug delivery in juveniles post-TBI. Application of our study also includes potentially targeting restoration of the BBB to reduce TBI-induced neuroinflammation and is grounded on an improved understanding of how BBB breakdown post-TBI may contribute to microglial activation. However, we highlight that the predictive relationships identified in this study should be viewed as a correlation, which does not necessarily indicate causation.

The findings of our study should be considered in the context of some limitations. First, our study was based on immunohistochemical findings. Given that we were measuring cell body size and microglial branch length, it must be noted that cells could be sliced through at any plane because of the nature of tissue processing for immunohistochemistry. Consequently, larger cells could appear smaller if they were sliced near the top or bottom of the cell span. To minimize the effect of this, we used Z-stacked images that spanned the depth of the tissue to best capture the cells. Second, our experimental subjects were solely male rats. Therefore, the future directions of this study include examination of the same outcome measures in female rats and testing for sex differences in the analyses. Third, for some of the comparisons in our study, effect sizes were small despite the differences being statistically significant; thus, additional studies need to be performed to fully understand the biological importance of some of our findings. Fourth, our model-based predictive relationships between IgG and microglial morphology, although clearly supported statistically, do not necessarily imply causal relationships.

It should also be noted that astrocytes are one of the main cellular components involved in BBB breakdown. However, we have previously shown that astrogliosis was minimal after diffuse TBI in juvenile rats^21^; thus, the current study focuses on microglia. It is possible that astrocytes contribute to the BBB dysfunction observed in this study. In the current study, we did not investigate our pathological outcome measures across time within each age group. There could be differences across age groups in the time course of transient BBB dysfunction and microglial activation, which warrants further investigation into these specific developmental age groups.

In conclusion, we found that IgG score predicted microglial activation in the brain of PND17 and PND35 rats. We also found some physiological differences in microglia morphology between PND17 and PND35 rodents, which may render PND17 rats more susceptible to microglia activation post-TBI. Together, these findings have important translational implications given that it may be a reliable method to predict the level of microglial activation in the brain post-TBI. Non-invasive measures of neuroinflammation encourage the development of personalized, age-specific treatments for TBI in the clinic.

## Supplementary Material

Supplemental data

Supplemental data

Supplemental data

Supplemental data

## Data Availability

The data sets supporting the conclusions of this article are publicly available in the Open Data Commons for Traumatic Brain Injury (ODC-TBI) at the following link: http://dx.doi.org/10.34945/F5Q304.

## Supplementary Material

Supplementary Information/Data

## Abbreviations Used

ABC: avidin-biotin complex
atm: atmospheres pressure
BBB: blood–brain barrier
CA: cornu ammonis
DAB: 3,3′-diaminobenzidine
DPI: days post-injury
IACUC: Institutional Animal Care and Use Committee
Iba1: ionized calcium-binding adaptor molecule 1
IgG: immunoglobulin-G
mFPI: midline fluid percussion injury
NGS: normal goat serum
NHS: normal horse serum
PBS: phosphate-buffered saline
PFA: paraformaldehyde
PND: post-natal day
TBI: traumatic brain injury

## References

[1] Serpa RO, Ferguson L, Larson C, et al. Pathophysiology of pediatric traumatic brain injury. Front Neurol 2021;12:696510; doi: 10.3389/fneur.2021.696510 34335452 PMC8319243

[2] Anderson V, Catroppa C, Morse S, et al. Functional plasticity or vulnerability after early brain injury? Pediatrics 2005;116(6):1374–1382; doi: 10.1542/peds.2004-1728 16322161

[3] Thurgur H, Pinteaux E. Microglia in the neurovascular unit: blood-brain barrier-microglia interactions after central nervous system disorders. Neuroscience 2019;405:55–67; doi: 10.1016/j.neuroscience.2018.06.046 31007172

[4] Monson KL, Converse MI, Manley GT. Cerebral blood vessel damage in traumatic brain injury. Clin Biomech (Bristol, Avon) 2019;64:98–113; doi: 10.1016/j.clinbiomech.2018.02.011 29478776

[5] Golding EM. Sequelae following traumatic brain injury. The cerebrovascular perspective. Brain Res Brain Res Rev 2002;38(3):377–388; doi: 10.1016/s0165-0173(02)00141-8 11890983

[6] Robertson CS, Contant CF, Gokaslan ZL, et al. Cerebral blood flow, arteriovenous oxygen difference, and outcome in head injured patients. J Neurol Neurosurg Psychiatry 1992;55(7):594–603; doi: 10.1136/jnnp.55.7.594 1640238 PMC489173

[7] Jenkins LW, Moszynski K, Lyeth BG, et al. Increased vulnerability of the mildly traumatized rat brain to cerebral ischemia: the use of controlled secondary ischemia as a research tool to identify common or different mechanisms contributing to mechanical and ischemic brain injury. Brain Res 1989;477(1–2):211–224; doi: 10.1016/0006-8993(89)91409-1 2702484

[8] Kratzer I, Liddelow SA, Saunders NR, et al. Developmental changes in the transcriptome of the rat choroid plexus in relation to neuroprotection. Fluids Barriers CNS 2013;10(1):25; doi: 10.1186/2045-8118-10-25 23915922 PMC3737068

[9] Kang R, Gamdzyk M, Lenahan C, et al. The dual role of microglia in blood-brain barrier dysfunction after stroke. Curr Neuropharmacol 2020;18(12):1237–1249; doi: 10.2174/1570159X18666200529150907 32469699 PMC7770642

[10] da Fonseca AC, Matias D, Garcia C, et al. The impact of microglial activation on blood-brain barrier in brain diseases. Front Cell Neurosci 2014;8:362; doi: 10.3389/fncel.2014.00362 25404894 PMC4217497

[11] Green TRF, Murphy SM, Rowe RK. Comparisons of quantitative approaches for assessing microglial morphology reveal inconsistencies, ecological fallacy, and a need for standardization. Sci Rep 2022;12(1):18196; doi: 10.1038/s41598-022-23091-2 36307475 PMC9616881

[12] Green TRF, Murphy SM, Moreno-Montano MP, et al. Reactive morphology of dividing microglia following kainic acid administration. Front Neurosci 2022;16:972138; doi: 10.3389/fnins.2022.972138 36248637 PMC9556904

[13] Morrison H, Young K, Qureshi M, et al. Quantitative microglia analyses reveal diverse morphologic responses in the rat cortex after diffuse brain injury. Sci Rep 2017;7(1):13211; doi: 10.1038/s41598-017-13581-z 29038483 PMC5643511

[14] Loane DJ, Kumar A. Microglia in the TBI brain: the good, the bad, and the dysregulated. Exp Neurol 2016;275 Pt 3(0 3):316–327; doi: 10.1016/j.expneurol.2015.08.018 26342753 PMC4689601

[15] Jiang Q, Wei D, He X, et al. Phillyrin prevents neuroinflammation-induced blood-brain barrier damage following traumatic brain injury. Front Pharmacol 2021;12:719823; doi: 10.3389/fphar.2021.719823 34744713 PMC8565465

[16] Barkauskas DS, Dixon Dorand R, Myers JT, et al. Focal transient CNS vessel leak provides a tissue niche for sequential immune cell accumulation during the asymptomatic phase of EAE induction. Exp Neurol 2015;266:74–85; doi: 10.1016/j.expneurol.2015.02.018 25708987 PMC4382434

[17] Neumann J, Riek-Burchardt M, Herz J, et al. Very-late-antigen-4 (VLA-4)-mediated brain invasion by neutrophils leads to interactions with microglia, increased ischemic injury and impaired behavior in experimental stroke. Acta Neuropathol 2015;129(2):259–277; doi: 10.1007/s00401-014-1355-2 25391494

[18] Richmon JD, Fukuda K, Maida N, et al. Induction of heme oxygenase-1 after hyperosmotic opening of the blood-brain barrier. Brain Res 1998;780(1):108–118; doi: 10.1016/s0006-8993(97)01314-0 9473615

[19] Bell MR. Comparing postnatal development of gonadal hormones and associated social behaviors in rats, mice, and humans. Endocrinology 2018;159(7):2596–2613; doi: 10.1210/en.2018-00220 29767714 PMC6692888

[20] Rowe RK, Harrison JL, Ellis TW, et al. Midline (central) fluid percussion model of traumatic brain injury in pediatric and adolescent rats. J Neurosurg Pediatr 2018;22(1):22–30; doi: 10.3171/2018.1.PEDS17449 29676680

[21] Green TRF, Murphy SM, Ortiz JB, et al. Age-at-injury influences the glial response to traumatic brain injury in the cortex of male juvenile rats. Front Neurol 2021;12:804139; doi: 10.3389/fneur.2021.804139 35111130 PMC8802670

[22] Hosseini AH, Lifshitz J. Brain injury forces of moderate magnitude elicit the fencing response. Med Sci Sports Exerc 2009;41(9):1687–1697; doi: 10.1249/MSS.0b013e31819fcd1b 19657303 PMC11421656

[23] Green TRF, Ortiz JB, Harrison JL, et al. Simultaneous cryosectioning of multiple rodent brains. J Vis Exp 2018;(139):58513; doi: 10.3791/58513 30295667 PMC6235249

[24] Schneider CA, Rasband WS, Eliceiri KW. NIH Image to ImageJ: 25 years of image analysis. Nat Methods 2012;9(7):671–675; doi: 10.1038/nmeth.2089 22930834 PMC5554542

[25] Young K, Morrison H. Quantifying microglia morphology from photomicrographs of immunohistochemistry prepared tissue using ImageJ. J Vis Exp 2018;(136):57648; doi: 10.3791/57648 29939190 PMC6103256

[26] Brooks ME, Kristensen K, van Benthem KJ, et al. glmmTMB balances speed and flexibility among packages for zero-inflated generalized linear mixed modeling. R J 2017;9(2):378–400; doi: 10.32614/Rj-2017-066

[27] Team RC. R: A language and environment for statistical computing. R Foundation for Statistical Computing: Vienna, Austria; 2022.

[28] Robitzsch A. Why ordinal variables can (almost) always be treated as continuous variables: clarifying assumptions of robust continuous and ordinal factor analysis estimation methods. Front Educ 2020;5:589965; doi: 10.3389/feduc.2020.589965

[29] Saber M, Murphy SM, Cho Y, et al. Experimental diffuse brain injury and a model of Alzheimer's disease exhibit disease-specific changes in sleep and incongruous peripheral inflammation. J Neurosci Res 2021;99(4):1136–1160; doi: 10.1002/jnr.24771 33319441 PMC7897258

[30] Hilbe JM. Modeling Count Data. Cambridge University Press: Cambridge, UK; 2014.

[31] Lüdecke D. ggeffects: Tidy Data Frames of Marginal Effects from Regression Models. J Open Source Softw 2018;3(26):772; doi: 10.21105/joss.00772

[32] Nakagawa S, Cuthill IC. Effect size, confidence interval and statistical significance: a practical guide for biologists. Biol Rev Camb Philos Soc 2007;82(4):591–605; doi: 10.1111/j.1469-185X.2007.00027.x 17944619

[33] Van Calster B, Steyerberg EW, Collins GS, et al. Consequences of relying on statistical significance: Some illustrations. Eur J Clin Invest 2018;48(5):e12912; doi: 10.1111/eci.12912 29438579

[34] Russell KL, Berman NE, Gregg PR, et al. Fish oil improves motor function, limits blood-brain barrier disruption, and reduces Mmp9 gene expression in a rat model of juvenile traumatic brain injury. Prostaglandins Leukot Essent Fatty Acids 2014;90(1):5–11; doi: 10.1016/j.plefa.2013.11.003 24342130 PMC3906920

[35] Onyszchuk G, He YY, Berman NE, et al. Detrimental effects of aging on outcome from traumatic brain injury: a behavioral, magnetic resonance imaging, and histological study in mice. J Neurotrauma 2008;25(2):153–171; doi: 10.1089/neu.2007.0430 18260798

[36] Greco T, Hovda DA, Prins ML. Adolescent TBI-induced hypopituitarism causes sexual dysfunction in adult male rats. Dev Neurobiol 2015;75(2):193–202; doi: 10.1002/dneu.22218 25066665

[37] Greco T, Hovda D, Prins M. The effects of repeat traumatic brain injury on the pituitary in adolescent rats. J Neurotrauma 2013;30(23):1983–1990; doi: 10.1089/neu.2013.2990 23862570 PMC3889497

[38] Bharadwaj VN, Rowe RK, Harrison J, et al. Blood-brainbarrier disruption dictates nanoparticle accumulation following experimental brain injury. Nanomedicine 2018;14(7):2155–2166; doi: 10.1016/j.nano.2018.06.004 29933022 PMC6177306

[39] Schmidt RH, Grady MS. Regional patterns of blood-brain barrier breakdown following central and lateral fluid percussion injury in rodents. J Neurotrauma 1993;10(4):415–430; doi: 10.1089/neu.1993.10.415 8145265

[40] Tanno H, Nockels RP, Pitts LH, et al. Breakdown of the blood-brain barrier after fluid percussion brain injury in the rat: Part 2: Effect of hypoxia on permeability to plasma proteins. J Neurotrauma 1992;9(4):335–347; doi: 10.1089/neu.1992.9.335 1291693

[41] McIntosh TK, Noble L, Andrews B, et al. Traumatic brain injury in the rat: characterization of a midline fluid-percussion model. Cent Nerv Syst Trauma 1987;4(2):119–134; doi: 10.1089/cns.1987.4.119 3690695

[42] Pardridge WM. Drug transport across the blood-brain barrier. J Cereb Blood Flow Metab 2012;32(11):1959–1972; doi: 10.1038/jcbfm.2012.126 22929442 PMC3494002

[43] Witcher KG, Bray CE, Chunchai T, et al. Traumatic brain injury causes chronic cortical inflammation and neuronal dysfunction mediated by microglia. J Neurosci 2021;41(7):1597–1616; doi: 10.1523/JNEUROSCI.2469-20.2020 33452227 PMC7896020

[44] Kim CK, Park JS, Kim E, et al. The effects of early exercise in traumatic brain-injured rats with changes in motor ability, brain tissue, and biomarkers. BMB Rep 2022;55(10):512–517; doi: 10.5483/BMBRep.2022.55.10.097 36104258 PMC9623238

[45] Witcher KG, Bray CE, Dziabis JE, et al. Traumatic brain injury-induced neuronal damage in the somatosensory cortex causes formation of rod-shaped microglia that promote astrogliosis and persistent neuroinflammation. Glia 2018;66(12):2719–2736; doi: 10.1002/glia.23523 30378170 PMC7542609

[46] Jin X, Ishii H, Bai Z, et al. Temporal changes in cell marker expression and cellular infiltration in a controlled cortical impact model in adult male C57BL/6 mice. PLoS One 2012;7(7):e41892; doi: 10.1371/journal.pone.0041892 22911864 PMC3404031

[47] Cao F, Yin A, Wen G, et al. Alteration of astrocytes and Wnt/β-catenin signaling in the frontal cortex of autistic subjects. J Neuroinflammation 2012;9:223; doi: 10.1186/1742-2094-9-223 22999633 PMC3544729

[48] Dos Santos SE, Medeiros M, Porfirio J, et al. Similar microglial cell densities across brain structures and mammalian species: implications for brain tissue function. J Neurosci 2020;40(24):4622–4643; doi: 10.1523/JNEUROSCI.2339-19.2020 32253358 PMC7294795

[49] Paolicelli RC, Bolasco G, Pagani F, et al. Synaptic pruning by microglia is necessary for normal brain development. Science 2011;333(6048):1456–1458; doi: 10.1126/science.1202529 21778362

[50] Schafer DP, Stevens B. Microglia function in central nervous system development and plasticity. Cold Spring Harb Perspect Biol 2015;7(10):a020545; doi: 10.1101/cshperspect.a020545 26187728 PMC4588063

[51] Chung WS, Barres BA. The role of glial cells in synapse elimination. Curr Opin Neurobiol 2012;22(3):438–445; doi: 10.1016/j.conb.2011.10.003 22036016 PMC3319527

[52] Lenz KM, Nelson LH. Microglia and beyond: innate immune cells as regulators of brain development and behavioral function. Front Immunol 2018;9:698; doi: 10.3389/fimmu.2018.00698 29706957 PMC5908908

[53] Liu J, Liu L, Wang X, et al. Microglia: a double-edged sword in intracerebral hemorrhage from basic mechanisms to clinical research. Front Immunol 2021;12:675660; doi: 10.3389/fimmu.2021.675660 34025674 PMC8135095

[54] del Zoppo GJ, Frankowski H, Gu YH, et al. Microglial cell activation is a source of metalloproteinase generation during hemorrhagic transformation. J Cereb Blood Flow Metab 2012;32(5):919–932; doi: 10.1038/jcbfm.2012.11 22354151 PMC3345906

[55] Walsh J, Tozer DJ, Sari H, et al. Microglial activation and blood-brain barrier permeability in cerebral small vessel disease. Brain 2021;144(5):1361–1371; doi: 10.1093/brain/awab003 34000009 PMC8874873

[56] Inkeaw P, Angkurawaranon S, Khumrin P, et al. Automatic hemorrhage segmentation on head CT scan for traumatic brain injury using 3D deep learning model. Comput Biol Med 2022;146:105530; doi: 10.1016/j.compbiomed.2022.105530 35460962

