# Supplementary material for: Blood–Brain Barrier Dysfunction Predicts Microglial Activation After Traumatic Brain Injury in Juvenile Rats
Source: Neurotrauma Rep. 2024 Feb 8;5(1):95–116. doi: 10.1089/neur.2023.0057 (PMC10890961; doi:10.1089/neur.2023.0057)

**FIGURE LEGEND**

**Figure S1: Microglial cell body perimeter in the hippocampus, hypothalamus, and motor cortex. (A)** Predicted microglial cell body perimeter across age-at-injury and **(B)** time post-injury in the hippocampus. **(C)** Predicted microglial cell body perimeter across age-at-injury and **(D)** time post-injury in the hypothalamus. **(E)** Predicted microglial cell body perimeter across age-at-injury in the motor cortex and **(F)** across time post-injury in the motor cortex. Results presented as the estimated conditional effects point estimates (dots) and their corresponding 95% confidence intervals (error bars) from generalized linear mixed effects models. Distributions of the raw data are represented by the background violins.


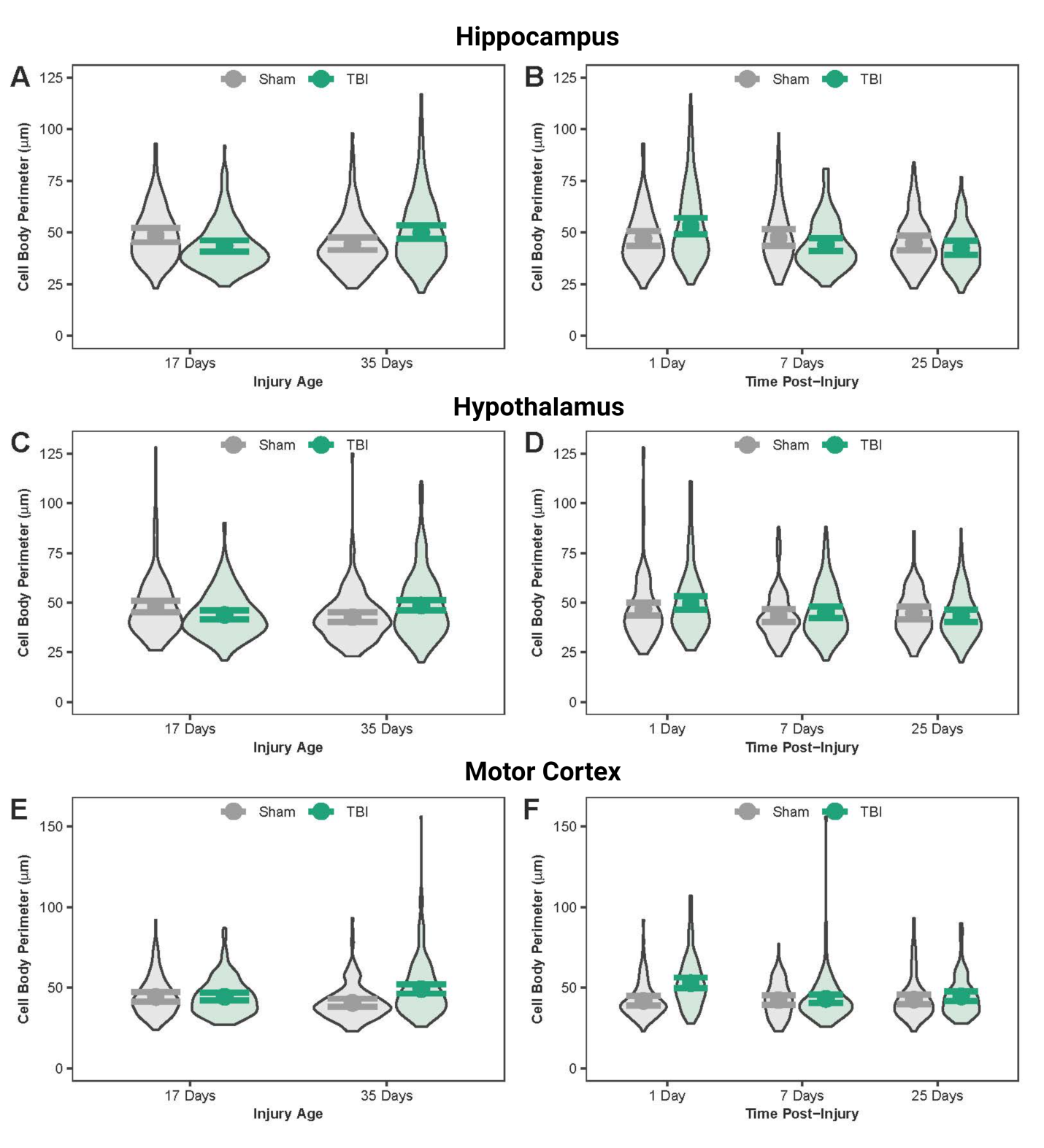

Supplement: Supplemental data [file Suppl_FigS1.docx]
