# Supplementary material for: Blood–Brain Barrier Dysfunction Predicts Microglial Activation After Traumatic Brain Injury in Juvenile Rats
Source: Neurotrauma Rep. 2024 Feb 8;5(1):95–116. doi: 10.1089/neur.2023.0057 (PMC10890961; doi:10.1089/neur.2023.0057)
Supplement: Supplemental data [file Suppl_TableS1.docx]

**TABLES**

**Table S1:** Estimated *p*-values and effect sizes for comparisons of microglial cell body perimeter in three brain regions among injury × age group combinations from generalized linear mixed models with negative-binomial error distributions. Statistical significance was achieved if *p* < 0.05, whereas biological significance was achieved if effect size > 0.20. Bold values denote biologically important effects.

| **Comparison** | **Hippocampus** | | **Hypothalamus** | | **Motor Cortex** | |
| --- | --- | --- | --- | --- | --- | --- |
|  | ***p*-value** | **Effect Size** | ***p*-value** | **Effect Size** | ***p*-value** | **Effect Size** |
| Sham PND17 vs. TBI PND17 | 0.09 | 0.01 | 0.13 | 0.005 | 0.99 | <0.001 |
| Sham PND17 vs. Sham PND35 | 0.28 | 0.004 | 0.03 | 0.01 | 0.24 | 0.004 |
| TBI PND17 vs. TBI PND35 | 0.01 | 0.01 | 0.03 | 0.01 | 0.06 | 0.004 |
| Sham PND35 vs. TBI PND35 | 0.06 | 0.01 | 0.01 | 0.01 | <0.001 | 0.01 |
