# Supplementary material for: Blood–Brain Barrier Dysfunction Predicts Microglial Activation After Traumatic Brain Injury in Juvenile Rats
Source: Neurotrauma Rep. 2024 Feb 8;5(1):95–116. doi: 10.1089/neur.2023.0057 (PMC10890961; doi:10.1089/neur.2023.0057)
Supplement: Supplemental data [file Suppl_TableS2.docx]

**Table S2:** Estimated *p*-values and effect sizes for comparisons of microglial cell body perimeter in three brain regions among injury × time post-injury group combinations from generalized linear mixed models with negative-binomial error distributions. Statistical significance was achieved if *p* < 0.05, whereas biological significance was achieved if effect size > 0.20. Bold values denote biologically important effects.

| **Comparison** | **Hippocampus** | | **Hypothalamus** | | **Motor Cortex** | |
| --- | --- | --- | --- | --- | --- | --- |
|  | ***p*-value** | **Effect Size** | ***p*-value** | **Effect Size** | ***p*-value** | **Effect Size** |
| Sham 1DPI vs. TBI 1DPI | 0.24 | 0.01 | 0.80 | 0.003 | <0.001 | 0.01 |
| Sham 1DPI vs. Sham 7DPI | 0.99 | <0.001 | 0.71 | 0.004 | 0.99 | <0.001 |
| Sham 1DPI vs. Sham 25DPI | 0.96 | 0.002 | 0.95 | 0.002 | 0.99 | <0.001 |
| TBI 1DPI vs. TBI 7DPI | 0.006 | 0.01 | 0.29 | 0.01 | <0.001 | 0.01 |
| TBI 1DPI vs. TBI 25DPI | 0.001 | 0.01 | 0.05 | 0.01 | 0.005 | 0.01 |
| Sham 7DPI vs. TBI 7DPI | 0.82 | 0.003 | 0.97 | 0.002 | 0.99 | <0.001 |
| Sham 7DPI vs. Sham 25DPI | 0.95 | 0.002 | 0.99 | 0.002 | 0.99 | <0.001 |
| TBI 7DPI vs. TBI 25DPI | 0.98 | 0.002 | 0.94 | 0.002 | 0.96 | 0.002 |
| Sham 25TBI vs. TBI 25DPI | 0.92 | 0.003 | 0.99 | 0.002 | 0.93 | 0.002 |
