# Supplementary material for: Blood–Brain Barrier Dysfunction Predicts Microglial Activation After Traumatic Brain Injury in Juvenile Rats
Source: Neurotrauma Rep. 2024 Feb 8;5(1):95–116. doi: 10.1089/neur.2023.0057 (PMC10890961; doi:10.1089/neur.2023.0057)
Supplement: Supplemental data [file Suppl_Information_or_Data.docx]

**SUPPLEMENTARY DATA**

There was no TBI effect on microglial cell body perimeters at PND17 (Supplementary Figure 1, see Supplementary Table 1 for *p*-values and effect sizes). There was an age effect on cell body perimeters in sham rats in the hypothalamus but not the hippocampus or motor cortex. There was also an age effect (PND35 rats had larger cell body perimeters than PND17 rats) in TBI rats in the hippocampus and the hypothalamus but not in the motor cortex. At PND35, TBI rats had larger cell body perimeters than sham rats in the hypothalamus and the motor cortex but not in the hippocampus. All comparisons of age-at-injury and cell body perimeter had low effect sizes.

The microglial cell body perimeter was greater in TBI rats compared to sham rats at 1DPI in the motor cortex but was not in the hippocampus or the hypothalamus (Supplementary Figure 1, see Supplementary Table 2 for corresponding *p*-values and effect sizes). There were no differences in microglial cell body perimeter between sham groups at any time point in any region examined. Microglial cell body perimeters were larger in TBI rats at 1DPI than 7DPI in the hippocampus and the motor cortex but were not in the hypothalamus, however, the estimated effect sizes were low. Microglial cell body perimeters were larger in TBI rats at 1DPI than at 25DPI in all regions examined. Microglial cell body perimeters were similar between TBI rats at 7DPI and TBI rats at 25DPI, regardless of age-at-injury. There was no TBI effect on cell body perimeter at 7DPI or 25DPI, suggesting that the change in cell body perimeter was transient. However, all differences in perimeter across time post-injury had low estimated effect sizes.
